# Grounding human-object interaction to affordance behavior in multimodal datasets

**DOI:** 10.3389/frai.2023.1084740

**Published:** 2023-01-30

**Authors:** Alexander Henlein, Anju Gopinath, Nikhil Krishnaswamy, Alexander Mehler, James Pustejovsky

**Affiliations:** ^1^Text Technology Lab, Faculty of Computer Science and Mathematics, Institute of Computer Science, Goethe University Frankfurt, Frankfurt, Germany; ^2^Situated Grounding and Natural Language Lab, Department of Computer Science, Colorado State University, Fort Collins, CO, United States; ^3^Lab for Linguistics and Computation, Department of Computer Science, Brandeis University, Waltham, MA, United States

**Keywords:** multimodal grounding, affordance detection, human-object interaction, habitat detection, multimodal datasets, neural models, transformers

## Abstract

While affordance detection and Human-Object interaction (HOI) detection tasks are related, the theoretical foundation of affordances makes it clear that the two are distinct. In particular, researchers in affordances make distinctions between J. J. Gibson's traditional definition of an affordance, “the action possibilities” of the object within the environment, and the definition of a *telic* affordance, or one defined by conventionalized purpose or use. We augment the HICO-DET dataset with annotations for Gibsonian and telic affordances and a subset of the dataset with annotations for the orientation of the humans and objects involved. We then train an adapted Human-Object Interaction (HOI) model and evaluate a pre-trained viewpoint estimation system on this augmented dataset. Our model, AffordanceUPT, is based on a two-stage adaptation of the Unary-Pairwise Transformer (UPT), which we modularize to make affordance detection independent of object detection. Our approach exhibits generalization to new objects and actions, can effectively make the Gibsonian/telic distinction, and shows that this distinction is correlated with features in the data that are not captured by the HOI annotations of the HICO-DET dataset.

## 1. Introduction

Introduced by Gibson in the 1970s, the concept of an “affordance” describes the functional and ecological relationship between organisms and their environments (Gibson, 1977). Gibson formulated the concept as a measure of what the environment “offers the animal” in terms of action possibilities of the object. In modern AI, particularly as it pertains to problems of perception in robotics (Horton et al., 2012) and grounding language to vision (McClelland et al., 2020), to say an object “affords” an action is to say that the object facilitates the action being taken with it. *Gibsonian* affordances are those behaviors afforded due to the physical object structure, and can be directly perceived by animals. For example, if a cup has a handle, it *affords* grasping and lifting by that handle. Pustejovsky, following from his Generative Lexicon theory (Pustejovsky, 1995) subsequently introduced the notion of a *telic* affordance, or behavior conventionalized due to an object's typical use or purpose (Pustejovsky, 2013). For example, a cup's conventional *purpose* is *for drinking from* and a book's is *for reading*. These conventionalized afforded behaviors are correlated with certain specific configurations between human and object; e.g., a chair must be upright with its seat clear to be sat in. These conditions (or *habitats*) form a precondition to the satisfaction of the intended use of the object; if those conditions are satisfied, the act of sitting on the chair will lead to the expected result of the chair supporting the human (i.e., its Telic qualia role according to Generative Lexicon theory). If not (e.g., the chair is upside down), the human will not be appropriately supported.

On the question of multimodal grounding, the computer vision and natural language processing (NLP) communities have drawn closer together, such that datasets originating in computer vision (e.g., Goyal et al., 2017; Damen et al., 2018; Boggust et al., 2019) now have demonstrated utility as benchmarks for NLP grounding tasks (e.g., Gella and Keller, 2017; Huang et al., 2020; Xu et al., 2020). One such popular challenge is grounding words to actions in images and video (e.g., Radford et al., 2021). As such actions often involve humans interacting with objects, datasets specialized to not just actions (running, jumping, walking, etc.) but to *human-object interaction* (HOI) have also proliferated in recent years (cf. Gupta and Malik, 2015; Krishna et al., 2016; Chao et al., 2018; Kim et al., 2021; Zou et al., 2021; Zhang et al., 2022).

Knowledge of how a human interacts with an object, however, is not always revealed through a single modality (language or image), and often even the alignment of multimodal annotations (e.g., bounding box and linguistic caption) does not adequately encode the actual HOI in a situation. For many HOIs, conventional descriptions used to caption them often fail to draw out significant aspects of the interactions that are important for creating visual embeddings. For example, it would be expected that an image with the caption “person driving a car” would share certain visual correlations with images of tools held in the hand, but there is usually no linguistic expression present in the caption to explicitly evidence that the driver is holding a steering wheel, etc.

Humans most often learn about affordances (e.g., “cups contain things,” “spoons are used for stirring”) by using objects or watching them in use (Tomasello, 2004); hence there is a natural alignment between affordance reasoning and various kinds of HOI tasks.

However, it must be noted that affordances and HOIs are not identical. Returning to Gibson's original formulation of the concept, he expands on it by stating that an affordance “implies the complementarity of the animal and the environment.” That is to say that the Gibsonian affordance, one afforded by an object's structure, is not just any action which can be taken with an object, but an action that is somewhat specific to that object and that agent in that environment. For example, the hollow geometry of a bottle *affords* containing liquids, while the opening *affords* releasing them. An image of a human drinking from a bottle, with it raised to the mouth, implies both the structure and the purpose of the bottle, even though neither is made explicit from the collocation of the object *bottle* and the action *drink_from*. It is this type of intentionality information, or identification of the relation between the object and human that is largely missing from grounded HOI datasets.

In this paper, we address the question of whether HOI models can distinguish the intentionality behind telic affordances from Gibsonian *exploitation* of an object.

Our novel contributions are as follows:

1. We present an augmentation of the HICO-DET (Chao et al., 2018) dataset that is annotated to distinguish Gibsonian from telic affordances at the visual and linguistic levels.
2. We developed AffordanceUPT, an adapted and modularized version of UPT (Zhang A. et al., 2021) that is trained over this novel data and can generalize to certain novel objects and actions.
3. We evaluate PoseContrast, a SOTA object orientation model, over the augmented dataset and find that PoseContrast tends to exhibit a strong bias toward the most frequent or default orientation, rather than the appropriate orientation for the action.

AffordanceUPT^1^ trained over the augmented HICO-DET dataset is able to accurately distinguish active intentional use from simple Gibsonian exploitation, and we find that the way objects cluster when the model is trained for the Gibsonian/telic distinction exposes additional correlations to the visual features of the specific images themselves.

## 2. Related work

There has been considerable interest in how encoding affordances might be used to improve the accuracy of HOI recognition and scene understanding models (Hassanin et al., 2021), as well as in downstream reasoning tasks in cognitive models of HOI or computational models of HRI. Psychological studies have shown that humans respond faster when objects are observed in canonical configurations (or *habitats* Pustejovsky, 2013) for their typical affordances (Yoon et al., 2010; Borghi et al., 2012; Natraj et al., 2015). Roboticists are particularly interested in affordances to model human-like interactions with objects, and work from that community has demonstrated that in order to successfully interact with an object, a robot need not know the object's name, but only perceive its function (Myers et al., 2015) or object affordances (Kim and Sukhatme, 2014; Saponaro et al., 2017). Affordances have also been recognized as implicating broader decisions for planning and inference (Horton et al., 2012; Antunes et al., 2016; Beßler et al., 2020).

The NLP community has made significant contributions in extracting object-oriented knowledge from language data. Multimodal datasets have been used to associate linguistic descriptions to visual information from action images, e.g., IMAGACT (Russo et al., 2013; Moneglia et al., 2018). Other research has explored integrating different descriptions of affordance information coming from language and visual datasets (Chao et al., 2015; Saponaro et al., 2017). Several approaches have identified objects' functional roles and factors involved with their creation using standard distributional techniques reflecting PPMI between action verbs and object types (Cimiano and Wenderoth, 2007; Yamada et al., 2007). These correlate with the *telic* (function) and *agentive* qualia (creation) a la Pustejovsky.

Recently it has become clear that not all modes of interacting with an object involve an affordance, while not all relevant object affordances are actually involved in the interaction the human is shown engaging in an image (Beßler et al., 2020; Hassanin et al., 2021). To address this, Pustejovsky (2013) defines a *habitat* as the precondition for an action to take place. Namely, a habitat is a conditioning environment or context that facilitates the enactment of an afforded behavior, such as how a bottle must be held to be drunk from. A primary component of habitats is object orientation, and therefore a potentially useful multimodal method for habitat detection is *pose detection*.

Pose detection has applications ranging from autonomous driving (Caesar et al., 2020), to robotics (Tremblay et al., 2018), and language grounding (Thomason et al., 2022). Consequently, available datasets are also diverse and specialized (more details in Section 3.3.2). Only recently has object orientation has been introduced into HOI Detection [e.g., D3D-HOI (Xu et al., 2021) or BEHAVE (Bhatnagar et al., 2022)]. So far, the focus has been mainly on human pose (e.g., Yao and Fei-Fei, 2010) or object size and positioning (e.g., Li et al., 2020).

## 3. An approach to detecting affordances

### 3.1. Theory

When humans identify and label objects, we not only perform a categorical type assignment (e.g., cup), but more often than not, we understand an entire set of object attributes as well as a network of relations concerning how the object participates in the situation under discussion. Many of these involve human-object interactions (HOIs), and our knowledge of things is predicated on an understanding of how we interact with them. Osiurak et al. (2017) provide a clear operationalization of this mechanical knowledge of affordances in the domain of tool use. In this domain, Norman (2002) divided Gibson's formulation into *physical* and *learned* affordances, and Young (2006) specified the notion of *functional* affordances. These specifications divide affordances into *hand-centered* and *tool-centered*, and the divisions map relatively straightforwardly to Gibson's affordances and Pustejovsky's telic affordances, but do not *per se* address the question of object orientation to the human.

For example, there is a conventional presupposition that the orientation of the cup exposes the concavity of the interior to enable the functioning of the cup (Freksa, 1992). Assuming that an object such as a cup, typed as a container, is asymmetric across the plane bisecting it horizontally, but otherwise a symmetrical cylindroid, it would appear that orientation information is critical for enabling the use or function of the object *qua* container. In fact, only when the cup's orientation facilitates containment can the function be “activated,” as it were. This references two notions that are critical for reasoning about objects and HOI generally: we encode *what* the function associated with an object is (its affordance) (Gibson, 1977), but just as critically, we also identify *when* it is active (its habitat) (Pustejovsky, 2013). Therefore, as given by Pustejovsky's original definition of the telic affordance, in this study we consider telic as a proper subset of the Gibsonian affordance, that overrides it; a telic affordance necessarily exploits the structural properties of the object, but does so in a way that *selects for a conventionalized configuration to activate a conventionalized function*.

To capture object type and human-object interaction potential, we adopt conventions used in the modeling language VoxML (Pustejovsky and Krishnaswamy, 2016), where habitats, including orientation, are modeled as preconditions on affordances, that is, the situational information about when/how an object is used. This allows modeling contextual and common-sense information about objects and events that is otherwise hard to capture in unimodal corpora, e.g., *balls roll because they are round*.

Hence the task of extracting dependencies between object habitats and affordances is consequential for tasks like automatic annotation of VoxML or Text-to-3D Scene applications (Chang et al., 2015). The current study focuses on adapting HOI models for affordance type classification using the Gibsonian/telic distinction and object orientation.

### 3.2. Annotation

#### 3.2.1. Image context annotations

**Figure 1:**
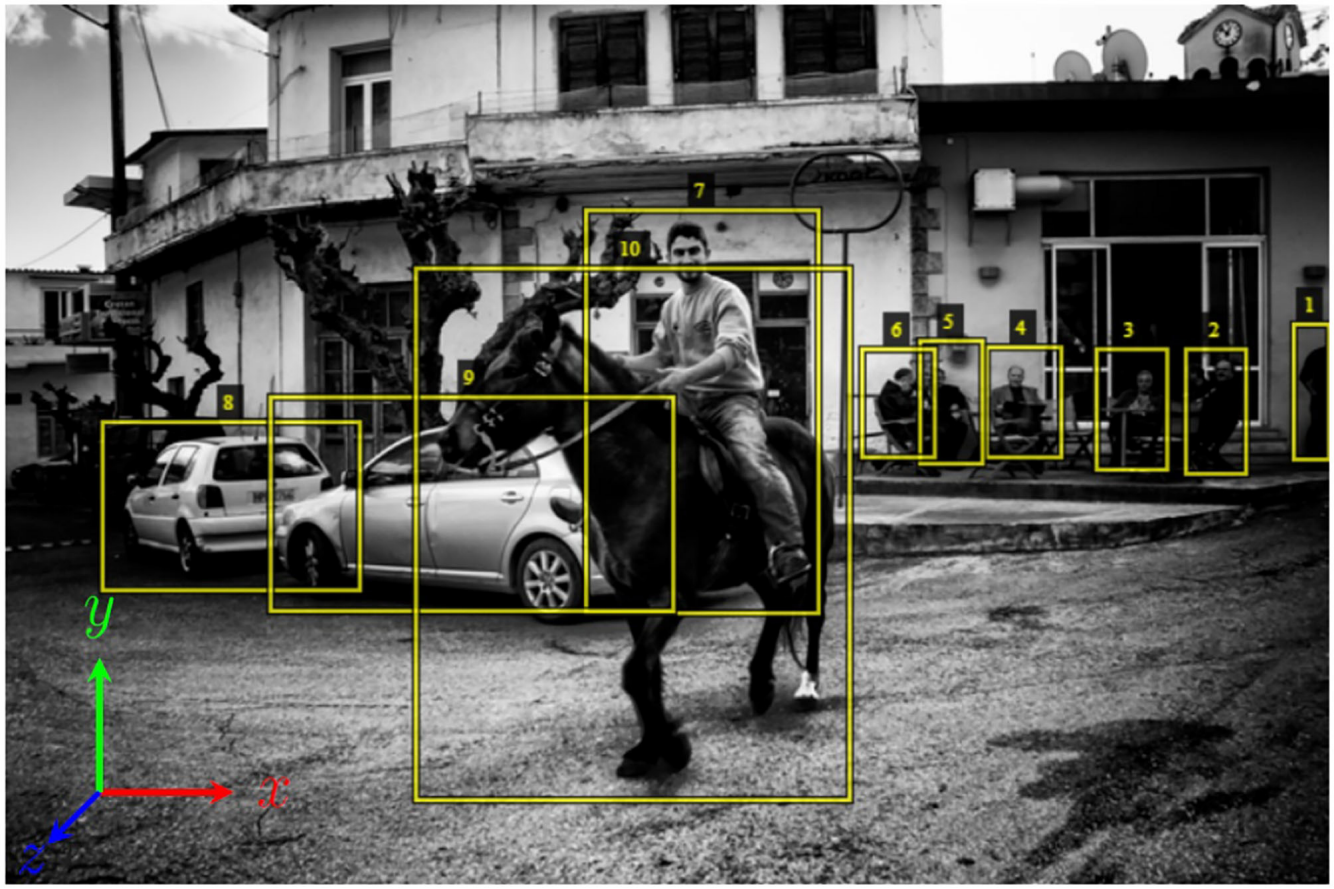
Example image context annotation. This HICO-DET image shows a telic affordance between horse (10) and person (7) and both with orientation: front(−1, 0, 1) up(0, 1, 0).

Our dataset consists of images taken from HICO-DET, a benchmark for HOI detection (Chao et al., 2018). Every image contains annotations for each HOI instance—bounding boxes for the humans and the objects with labels for the interactions. We annotated 120 images taken from 10 object categories for a total of 1,200 images. The 10 object categories are *apple, bicycle, bottle, car, chair, cup, dog, horse, knife*, and *umbrella*, chosen for being representative of the full set of HICO-DET object categories, which includes animals, vehicles, and household objects. Using a modification of the VIA tool (Dutta et al., 2016; Dutta and Zisserman, 2019) as shown in Figure 1, each image was annotated for the *action, affordance* class (Gibsonian/telic), and direction of *front* and *up* orientation of the objects therein. *Action* and *affordance* were annotated for all the relevant humans in an image, and orientation fields *up* and *front* were annotated for both the objects and the humans. Additionally, fields *is_part_of* and *changes?* were used to track whether an item being annotated was part of another annotated item and whether any changes were made in the annotations (new object or action) from those specified in the HICO-DET dataset, respectively.

The possible options for the field *affordance* are *None, Gibsonian* (G) and *telic* (T). The affordance is marked as G when the action performed is by virtue of the object's structure and T if by virtue of the object's conventionalized use or purpose (see Section 3.1). The fields *action* and *obj name* are chosen from the list of actions and object names respectively provided in the HICO-DET dataset. Front and upward orientations are selected from the world orthogonal axes [*x, y, z*]. When viewing an image face-on, +*x* is to the right of the screen, −*x* is to the left, +*y* is upward and −*y* is downward, while +*z* extends out of the screen toward the annotator and −*z* is pointing away from them into the screen. This assumes a standard right-hand coordinate system as shown in Figure 1. Axes can be combined. If the front of the human or object faces both leftward and forward (out of the image), then the *front* orientation is −*x*+*z*, and +*x*+*z* if turned halfway toward the right. If no clear front or top was apparent (e.g., for a ball), it was annotated as [0, 0, 0]. In this paper we denote orientation using the notation *front*_*up* with each vector represented as (*x, y, z*). The horse in Figure 1 would be denoted [−1, 0, 1]_[0, 1, 0], because its forward vector is facing toward the left (−*x*) and out of the image (+*z*) while its intrinsic up vector is pointing up (+*y*).

These annotations were later used to evaluate Object Pose Detection (see Section 3.3.2) and to evaluate the overall Habitat Extraction approach (Section 4.4).

#### 3.2.2. Text annotations

Each of the 600 object-verb pairs in the HICO-DET dataset were also annotated with the affordance (G for Gibsonian or T for telic). Table 1 shows a few examples. In HICO-DET, people and objects are often associated with multiple verbs (e.g., a person sits, rides, and races a motorcycle). If one action of such a set has been defined as telic, we define the action as a telic affordance; this is because telic affordances are supervenient on any existing Gibsonian affordances, hence they can formally be said to take precedence over any accompanying implicated Gibsonian affordance. Since telic affordances are necessarily more specific and informative than Gibsonian affordances, they are considered to subsume them, and therefore defining the same affordance as both telic and Gibsonian would be redundant—see Section 3.1 for more information.

**Table 1:** A small subset of text annotations.

**Object**	**Action**	**Affordance**
Bicycle	Ride	T
Bicycle	Hold	G
Bottle	Hold	G
Bottle	Drink_with	T
Cow	Milk	T
Cat	Feed	T
Banana	Carry	G
Skis	Pick_up	G
Knife	Cut_with	T

G stands for Gibsonian and T for telic.

These text-only annotations have the advantage of rapidly generating data for training HOI models, while lacking some additional contextual information that may be provided by an image, as in Section 3.2.1. These annotations were later used to train and evaluate the AffordanceUPT model (see Section 3.3.1).

Image and text annotation were each performed by different people. The calculated IAA is listed in the Supplementary material.

### 3.3. Models

#### 3.3.1. Human-object interaction

We adapted the UPT (*Unary-Pairwise Transformer*; Zhang et al., 2021a) model as the basis for Gibsonian/telic affordance classification. UPT is a two-step transformer-based (Vaswani et al., 2017) HOI classifier and its authors demonstrate that it is comparatively performant and memory efficient compared to other state-of-the-art HOI models (e.g., Tamura et al., 2021; Zhang et al., 2021b). In the first step, it determines all relevant entities and in the second step their relations (in contrast to single-task models, where entities and relations are considered together in multi-task learning; Zhang A. et al., 2021). UPT is therefore composed of two parts: a *cooperative transformer*, which operates on *unary tokens* to generate a representation of entities, and a *competitive transformer*, which subsequently operates on *pairwise tokens* to represent their relations.

Moreover, the two-step approach enables the analysis of both representations of objects (*unary tokens*) and of their interactions (*pairwise tokens*) (see Section 4).

To utilize UPT for affordance detection, we changed the classification from a variable number of verbs to a two-label Gibsonian/telic classification. We also modularized UPT to make the affordance detection independent of object detection based on DETR (*Detection Transformer*; Carion et al., 2020), which uses ResNet (He et al., 2016) as a backbone. That is, we replaced the pre-trained, inflexibly implemented DETR variant (supporting 80 object types) with a modular variant from Huggingface^2^ (supporting 90 object types) and froze all DETR/ResNet weights. However, nothing fundamental was changed in the underlying architecture. This makes our UPT variant independent of the object detection module so that it can be replaced by models that support other object types. We will refer to the model as **AffordanceUPT** in the remainder of this paper. The performance of AffordanceUPT on unknown objects and actions is also part of our evaluation (see Section 4.1). Our approach to affordance detection shows how methods such as UPT can be applied to this and related tasks in multimodal semantics.

#### 3.3.2. Object pose estimation

To estimate object orientation, we use PoseContrast (Xiao et al., 2021). This model has the advantage of not requiring additional information such as CAD references or class information, while still providing strong results (cf. Xiao et al., 2019; Dani et al., 2021; Nguyen et al., 2022). We retrained the model on the ObjectNet3D dataset (Xiang et al., 2016), which is still one of the largest datasets for this task with 100 object categories and over 90,000 images. Other common datasets are still very limited in their domain or object categories (see also Supplementary material).

#### 3.3.3. Training

AffordanceUPT was trained for 20 epochs on 2 GeForce RTX 8000 devices with a batch size of 8 per GPU—an effective batch size of 16. Hyperparameter optimization was performed using W&B (Biewald, 2020). The resulting parameters are listed in the Supplementary material. The respective HICO-DET dataset, annotated with Gibsonian/telic labels as described in Section 3.2.2, served as training and test data. Images without Gibsonian/telic text annotations were removed, resulting in a dataset size of 33,593 training images and 8,527 testing images. In addition to training with the regular HICO-DET split, we also trained variants to evaluate generalization to unknown objects and actions (see Section 4.1).

PoseContrast was trained on one GeForce RTX 8000 with default parameters. Different hyperparameters and additional methods of augmenting the training data were tested, but did not result in significant improvements.

## 4. Evaluation and analyses

### 4.1. Evaluation of AffordanceUPT

**Figure 2:**
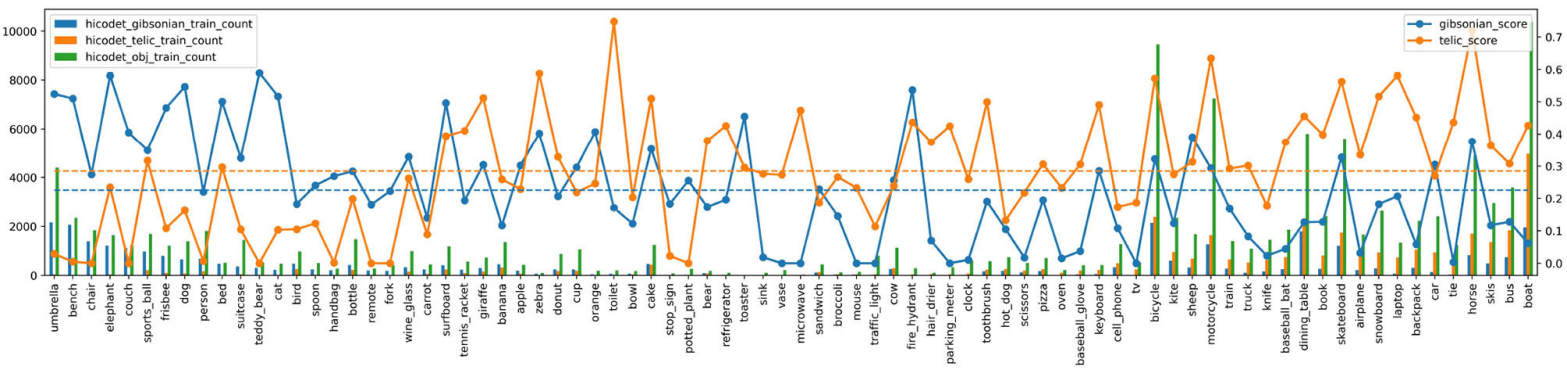
AffordanceUPT evaluation regarding object types and training data size. The bottom axis lists the object labels. The left axis and associated bar graphs show the number of *Gibsonian* (blue), *telic* (orange), and general object occurrences (green) in the HICO-DET training subset. The right axis and corresponding line graph show the mAP for each object. Dashed lines denote overall mean values for the two affordance types. The objects are sorted by the ratio between G and T training samples.

For the evaluation of AffordanceUPT see Table 2 and Figure 2. The results show that HOI models can also be used for affordance detection with a few adjustments, as shown in the example of UPT. The mAP values are within ~1–5 mAP) of HOI detection on the regular HICO-DET dataset (cf. Hou et al., 2021b; Tamura et al., 2021; Zhang et al., 2021a). The differences are for a few reasons:

1. The distributions of our target classes are much more complex, subsuming multiple diverse actions;
2. HICO-DET has separate bounding boxes for each action, and these can vary widely, resulting in multiple boxes for the same object or person;
3. Not every affordance in HICO-DET is always annotated but AffordanceUPT detects them anyway;
4. Our object detection model is not trained on HICO-DET, so there can be major deviations for the boundary boxes that cannot be merged.

**Table 2:** AffordanceUPT results on the Gibsonian/telic text annotated HICO-DET dataset where the first line is our default AffordanceUPT model trained and evaluated on the regular HICO-DET split.

	**Training data**	**Test data**	**mAP x 100**
	*HICO-DET Train*	*HICO-DET Test*	27.58
Object	*HICO-DET Merged* w/o bicycle	*HICO-DET Merged* bicycle	35.74
	*HICO-DET Train*	*HICO-DET Test* bicycle	46.69
	*HICO-DET Merged* w/o car	*HICO-DET Merged* car	20.44
	*HICO-DET Train*	*HICO-DET Test* car	33.54
Verb	*HICO-DET Merged* w/o wield	*HICO-DET Merged* wield	32.99
	*HICO-DET Train*	*HICO-DET Test* wield	37.23
	*HICO-DET Merged* w/o drive	*HICO-DET Merged* drive	21.40
	*HICO-DET Train*	*HICO-DET Test* drive	26.05
Obj+verb	*HICO-DET Merged* w/o book or read	*HICO-DET Merged* book and read	24.11
	*HICO-DET Train*	*HICO-DET Test* book and read	31.46
	*HICO-DET Merged* w/o car or drive	*HICO-DET Merged* car and drive	15.63
	*HICO-DET Train*	*HICO-DET Test* car and drive	22.63

*HICO-DET Merged* stands for the data set combined from training and test data. *w/o* denotes models that have been trained without the respective object/verb.

A few examples can be found in the Supplementary material. These do not significantly affect training and inference, but are reflected in the evaluation score since the problem primarily concerns the boundary boxes and not the affordance label itself. We deliberately decided against alternative datasets like V-COCO (Lin et al., 2014; Gupta and Malik, 2015) or VisualGenome (Krishna et al., 2016), as V-COCO has a very limited set of verbs (26) and VisualGenome is too unstructured for now.

To evaluate AffordanceUPT on novel objects, we select a few specific examples, specifically: the nouns *bicycle* and *car*, the verbs *wield* and *drive*, and the HOIs *book*+*read* and *car*+*drive* (see Table 2). In Table 2, *HICO-DET Merged w/o bicycle (first column)* denotes a dataset created from combining train and test images without bicycles in them (used for training), whereas *HICO-DET Merged bicycle (second column)* has combined train and test images with bicycles in them (used for testing) and *HICO-DET Merged Test bicycle (second column)* denotes images from the test set with bicycles in them (used for testing). *HICO-DET Train* and *HICO-DET Test* denotes the regular train and test set respectively. We re-split HICO-DET such that for each example, the test set comprised all images containing the example, while the training data comprised all remaining images (i.e., for *car*+*drive*, images of boats being driven or cars being washed were omitted from both training and evaluation). These results were then compared against the results of the normal AffordanceUPT model on the objects/verbs in the regular HICO-DET test dataset.

Our results show that AffordanceUPT can detect affordances on novel objects, albeit with an appreciable drop in mAP (e.g., ~10–13%). The effect is less strong for unknown actions such as *driving* (only a drop of around 5%). AffordanceUPT can even generalize to some extent to novel objects and actions (e.g., detecting that driving a car is a telic affordance, despite never seeing a car *or* a driving action). Meanwhile, regular HOI models generalize only on unknown HOI combinations (e.g., Shen et al., 2018; Hou et al., 2021b) or on unknown objects (e.g., Wang et al., 2020; Hou et al., 2021a), not both.

Because each re-split requires retraining, the evaluation could not be carried out for all combinations due to runtime reasons. However, the tendencies are clearly apparent.

The generalization on display here is only made possible by our abstraction to the two affordance types that point to specific kinds of action classes that can be contained under the same label. This means affordance detection supports a higher level of generalization due to greater abstraction. Further, the ability to distinguish between the two affordance types, telic and Gibsonian means that the model can also identify when an object is being actively used, since telic affordance indicates active usage and Gibsonian indicates mere interaction with the object. This makes affordance detection interesting for applications where the exact action does not need to be detected, but a distinction of intentional or active use is sufficient.

Such situations could be, for example:

i) Monitoring an object's active usage time. For example, a knife can be held in several different ways. But, to use a knife for cutting something, the blade of the knife needs to be pointing down toward the object. Using these criteria, we can estimate when a knife is likely to be dull from continued use and needs sharpening.
ii) For autonomous driving. For example, whether a pedestrian is distracted by the active use of an object and therefore more caution is required (Papini et al., 2021).
iii) Language grounding applications, such as grounding for robotics (Ahn et al., 2022). For example, aiding a robot in distinguishing between interactive and non-interactive gestures (Matuszek et al., 2014). A robot can learn to identify that in order to grasp an object, the anthropomorphic hands/grippers should be positioned above the object before attempting the grasp. The grasp would depend on the specific task one is trying to execute, and whether that task exploits a Gibsonian affordance or a telic one. The orientation of the object is also important in some cases—e.g., to hold a cup for the purpose of pouring (telic) something from the cup to a bowl—in this case, the top of the cup should be tilted toward the bowl, and orientation is one of the object attributes we annotated (Section 3.2.1).
iv) Visual question answering (Antol et al., 2015). For example, to generate better answers to the question “What is the person doing?”. Consider an image of a human-object interaction where a person is holding an umbrella. Based on the intentionality of the interaction, the answer could be “the person is holding the umbrella upright to shield himself from the rain” vs. “the person is carrying the umbrella with him in case it rains.”
v) Image captioning (Nguyen et al., 2021)—specifically in cases where the verb implies one kind of affordance but the image indicates the other. For example, if an image of a “riding” affordance shows a passenger riding in a car beside the driver with hands on the steering wheel, our model would still be able to detect that the car is being used for an intentional “driving” action. In Figure 5, we show distinct clusters of car “riding” action images where the driver's hands are visible and where they are not.

### 4.2. Evaluation of PoseContrast

**Figure 3:**
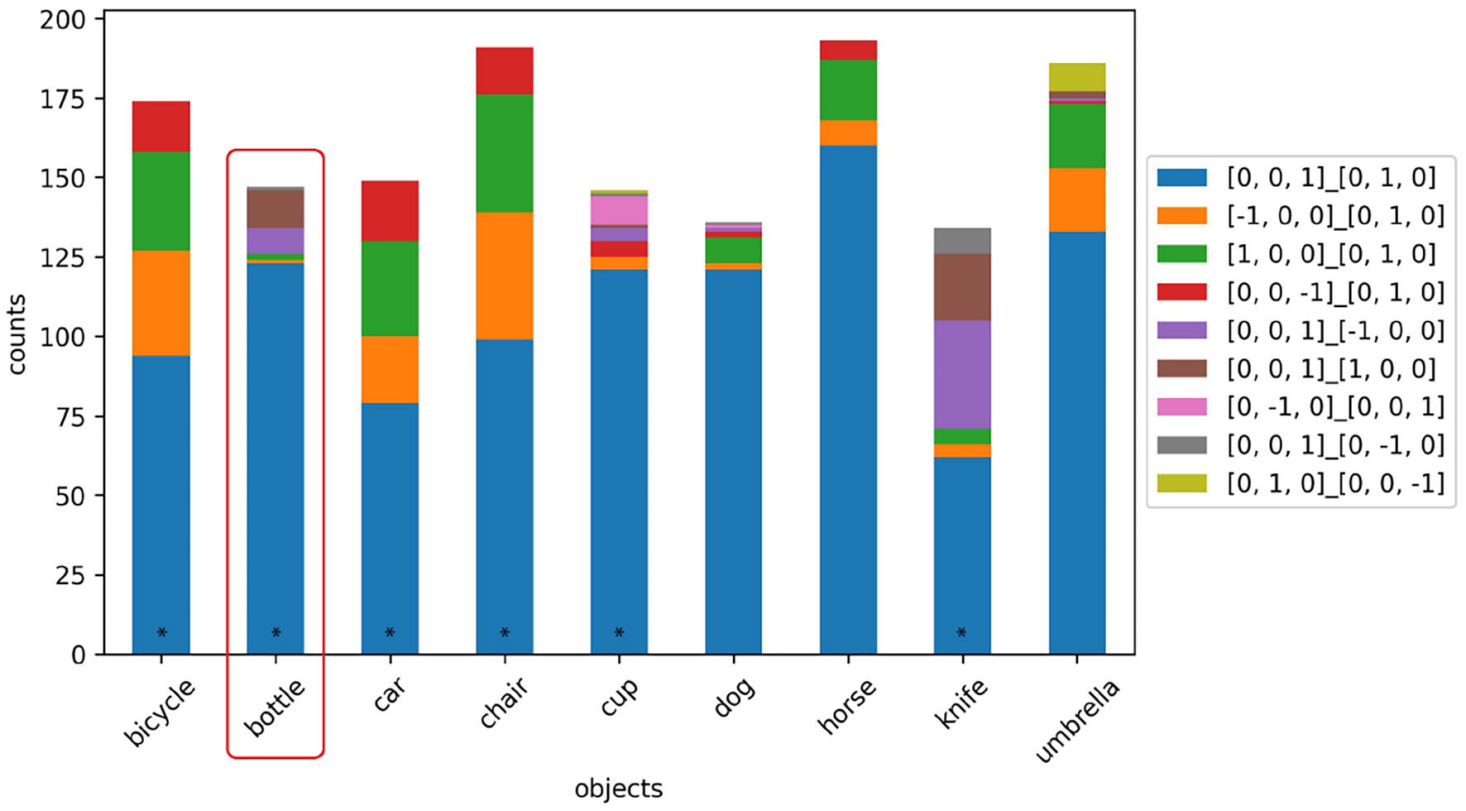
PoseContrast orientation predictions on the 1,200 annotated HICO-DET images for 9 object classes. Predicted orientations with a frequency of < 5 were filtered out. *Marks objects that are also in ObjectNet3D.

**Figure 4:**
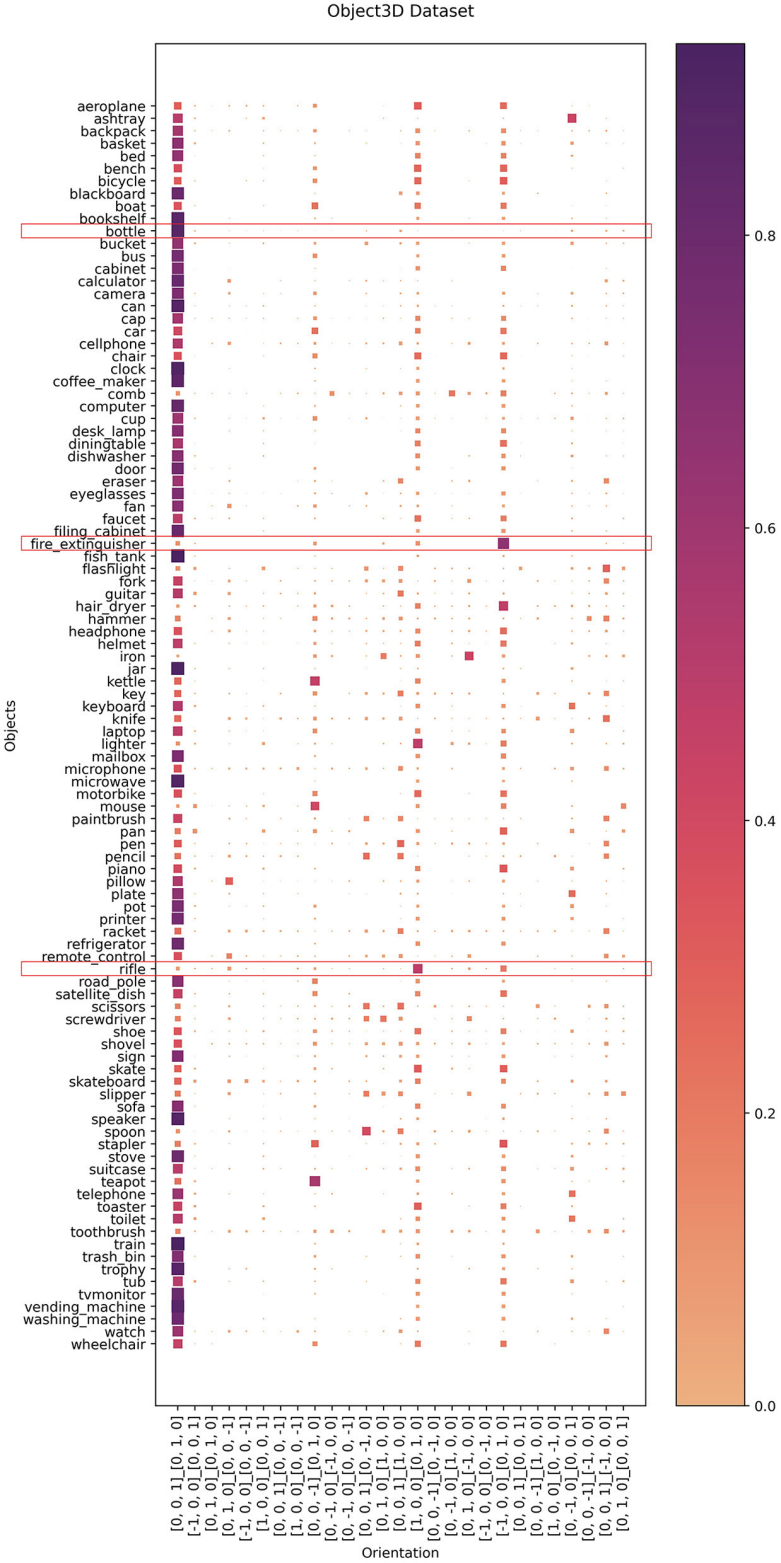
ObjectNet3D dataset mapped to main orientations. Scores are weighted for every object. An interesting example (red box) is “bottle,” which occurs almost exclusively in an upright position in the dataset. Other interesting examples include “fire extinguisher” and “rifle,” which also exist in the dataset in stereotypical pose (cf. Barbu et al., 2019), but which for these objects means that the front of the object points to the side of the image.

We used the 1,200 image annotations of HICO-DET from Section 3.2.1 to evaluate PoseContrast. Since PoseContrast outputs object rotation as Euler angles, but the annotations indicate the major axis orientation, the PoseContrast output was mapped to these axes. The evaluation scores thus describe the accuracy with which the objects were aligned with the correct major axes. We compare PoseContrast with two baselines: one, in which the object is always predicted to be facing forward and upright ([0, 0, 1]_[0, 1, 0]), and a second, which always predicts the most frequent orientation in the HICO-DET annotations (*Most Frequent*). The results are listed in Table 3. PoseContrast appears to generalize very poorly on the HICO-DET dataset. Notably, the default orientation [0, 0, 1]_[0, 1, 0] is predicted for almost all objects (see Figure 3), including for object classes in the training set. Examining the ObjectNet3D dataset, we find that it almost exclusively contains objects in this orientation (e.g., upright bottles, forward-facing TVs), rather than in orientations where they are manipulated by humans (i.e., Gibsonian or telic affordances) (see Figure 4). Rotating the image serves as an augmentation method during training but is of limited use. For example, if only side views of weapons are available, it is impossible to generate views from the front or back. We also tried additional augmentation methods such as blur filters and dpi variations, but they did not produce significantly better results. Further analyses can be found in the Supplementary material.

**Table 3:** PoseContrast results on the image annotated HICO-DET dataset.

**Model**	**Apple**	**Bicycle**	**Bottle**	**Car**	**Chair**	**Cup**	**Dog**	**Horse**	**Knife**	**Person**	**Umbrella**
[0,0,1]_[0,1,0]	0.18	0.13	0.57	0.19	0.27	0.72	0.20	0.21	0.01	0.40	0.73
Most frequent	0.65	0.41	0.57	0.38	0.31	0.72	0.21	0.41	0.18	0.40	0.73
PoseContrast	0.83	0.44	0.67	0.51	0.58	0.75	0.31	0.25	0.08	0.44	0.67

The object names in black are also represented in ObjectNet3D. The first two rows represent the baselines. The top row shows the accuracy for a hypothetical model that always predicts the orientation vector [0,0,1]_[0,1,0] (oriented upright with respect to the viewer), and the second row shows the accuracy if the most common orientation for the object in the dataset is always predicted.

### 4.3. Analysis of AffordanceUPT tokens

**Figure 5:**
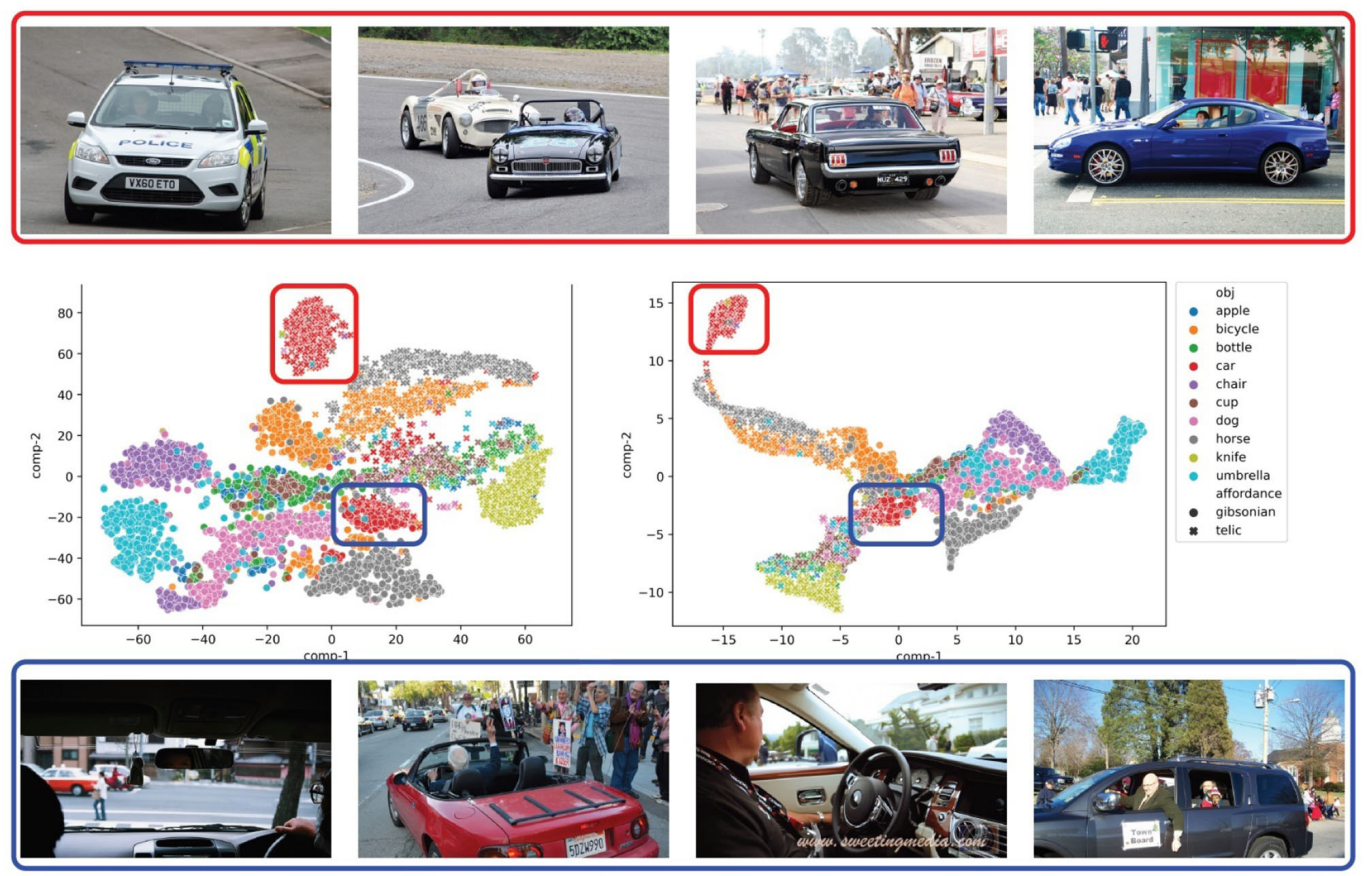
AffordanceUPT token-pair visualization using t-SNE **(Left)** and PaCMAP **(Right)**. The vehicle images above and below are “ride” images from the HICO-DET dataset and are classified as telic by the model. The images in the **top row** are in the red cluster and the images in the **bottom row** are in the blue cluster.

To show how AffordanceUPT distinguishes between Gibsonian and telic affordances, in Figure 5 we visualize the token-pair representations for the 10 test categories using t-SNE and PaCMAP (Wang et al., 2021). We see that objects that are interacted with in a similar way and have similar affordances appear closer together. For example, the occurrences of *bottle* and *cup* (i.e., containers to drink liquids from) are strongly overlapping. Also, *bicycles* and *horses*, both rideable, are placed close to each other when considering telic affordances. Gibsonian interactions with *horses*, on the other hand, are closer to those with *dogs* (and do not occur in the large Gibsonian *bicycle* cluster). In addition, all objects (e.g., *apple, bottle, cup, knife*) that imply interaction primarily with the hand are in the same region, which includes some images of cars (blue marked cluster), an initially rather unintuitive observation. But a look at the different images for “ride” in the two car clusters, explains this. In the blue cluster (closer to the hand-held objects), the interactions of the hand with the car (e.g., steering wheel) are more clearly visible, while in the red cluster the people (and therefore hands) are less visible, and the images focus more on the entire car and the actual “driving” aspect. The same apparent HOI action class (in this case, “ride”), as given by the original labels in HICO-DET, in fact divides into distinct clusters based simply on how the model is trained to represent the two-way affordance type distinction (Gibsonian and telic). This directly reflects one of the potential application domains of this work mentioned in Section 4.1. Such information is essential for accurately grounding visual human-object interactions to language, and thus leads us back to the motivation from the introduction: information like this is linguistically redundant (e.g., “man driving a car with his hands on the steering wheel” is non-informative because driving—at present—presupposes steering). Only with image examples do these features make semantic sense. This work paves the way for systematically extracting such visual information and linking it to language. Visualizations of the *unary tokens* can be found in the Supplementary material.

### 4.4. Automated habitat annotation

**Figure 6:**
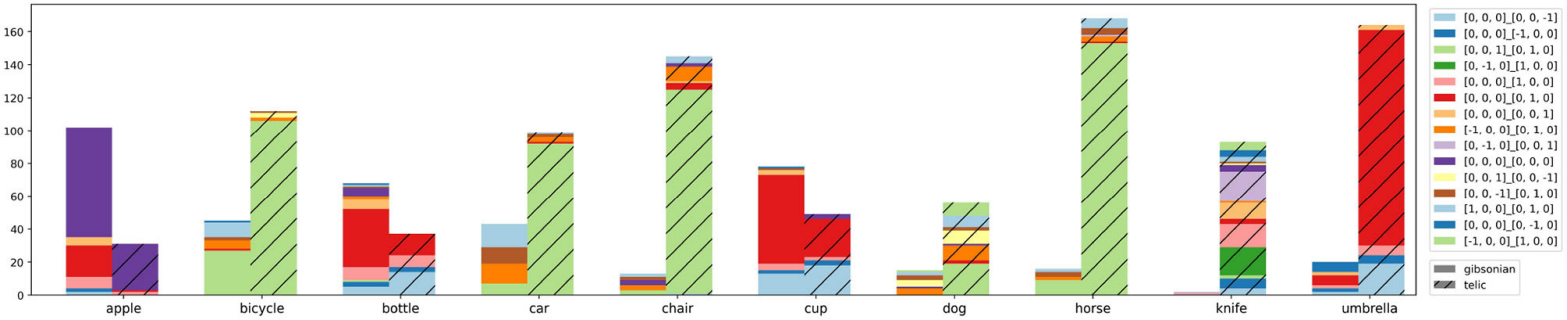
Habitats based on the 1,200 image annotations. The colors here represent the relative alignments in relation to the person.

As automatic determination of object orientation is still limited, we analyze habitats based on our HICO-DET image annotations. We converted object orientations in world space to be relative to the interacting person (e.g., the person's front is now +*z*). In Figure 1, the horse would have the orientation [0, 0, 1]_[0, 1, 0], since it is oriented in the same direction as the person. Figure 6 depicts the resulting statistics, and shows the relationship between affordance and object orientation as a habitat condition. The orientation of objects like *bicycles, cars, chairs, horses*, and *dogs* is relatively independent of their affordances, but these objects are often aligned in the same way as the person in the case of a telic scenario. Bottles and cups, on the other hand, show a strong relative increase in orientation to [0, 0, 0]_[0, 0, −1], indicating that the object's upward is oriented opposite to the person's front (typical orientation when drinking). Knives, on the other hand, can be held in any orientation, however the majority of orientations (green segment plus orange segment) indicate that knives are often held with the blade facing down, away from the person.

Figure 6 shows the interdependence of affordance and orientation (as a subcondition of habitat): affordances presuppose certain orientations, and conversely, certain object orientations make certain affordances possible in the first place. Therefore, both variables should be considered in relation to each other (in relation to HOI as a whole) and not as independent phenomena.

## 5. Discussion and conclusions

We presented AffordanceUPT, an adaptation of UPT to distinguish between Gibsonian and telic affordances. With some augmentations to HICO-DET and modularization of UPT, we can alter a powerful HOI detection model to detect distinctions in affordances specifically. This greater level of abstraction lends itself to generalization that was not possible before from a forced-choice HOI detection model, and in the process we uncovered properties of the data that have important implications for grounding images to language.

Our model performs affordance detection even on novel objects. We highlight the limitations of habitat (orientations) modeling in existing datasets using PoseContrast. Further, we also visualize the Gibsonian/telic distinction which highlights interesting HOI attributes.

We found that how AffordanceUPT clusters objects indicates what can be detected by automatic entity and intention detection. Such distinctions are useful for (semi) automatically populating a multimodal representation like VoxML (Pustejovsky and Krishnaswamy, 2016) by inferring possible affordances for an object and their preconditions. AffordanceUPT also shows promise in generalization for novel objects and actions, meaning it could also infer partial information about novel objects or events for such a representation.

### 5.1. Future work

In future work, we plan a comprehensive analysis of AffordanceUPT's performance on novel entities with respect to which training conditions must be fulfilled for the model to classify which attributes.

Results and interpretations like those in Figure 5 were performed on a manageable subset of data. Further analysis could determine how our method scales when dealing with big data, using automated analysis techniques. In addition, since annotations were only performed on a subset of the HICO-DET dataset, one item of future work is to enlarge the dataset, including using crowdsourcing techniques.

Now that we have established the validity of the AffordanceUPT Gibsonian/telic discrimination approach, next steps also include doing cross-dataset validation, such as training on HICO-DET and evaluating on V-COCO, to further establish generalizability or the requirements for generalizable Gibsonian/telic discrimination.

The division into Gibsonian and telic affordances can also be further refined. For example, the act of “repairing a car” is not a telic affordance, but an act of *maintaining* telic functionality.

Successful habitat detection depends on improving performance on the remaining challenge of object orientation detection. In the future, we plan to test our approach on a larger scale and expand the dataset for this purpose. This may involve combining AffordanceUPT with grounded language models e.g., CLIP (Radford et al., 2021).

## Data availability statement

The data is available under: https://github.com/VoxML/affordance-annotation.

## Author contributions

AH, AG, NK, AM, and JP contributed to the conception, design of this work, and wrote sections of the manuscript. AH developed, trained, and analyzed the models. AG developed the annotation tool, textual, and image annotations. JP gave textual annotations. NK and JP gave the necessary theoretical background for affordances and HOI. NK, AM, and JP gave regular feedback and suggestions for improvements. All authors contributed to manuscript revision, read, and approved the submitted version.

## Footnotes

^1^All models and annotations corresponding for this work can be found under https://github.com/VoxML/affordance-annotation.

^2^ https://huggingface.co/facebook/detr-resnet-50
