# Supplementary material for: Grounding human-object interaction to affordance behavior in multimodal datasets
Source: Front Artif Intell. 2023 Jan 30;6:1084740. doi: 10.3389/frai.2023.1084740 (PMC9923013; doi:10.3389/frai.2023.1084740)
Supplement: Supplementary file 1 [file Data_Sheet_1.PDF]

Supplementary Material

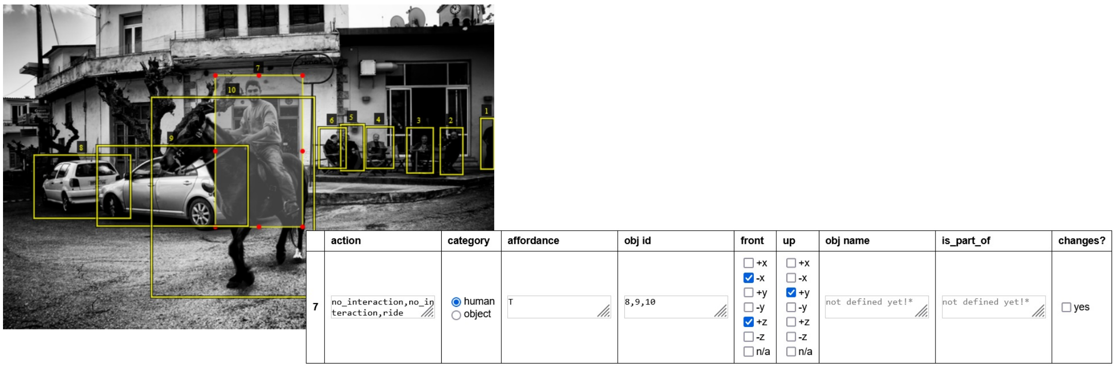

Figure 1a. Example of human annotation.

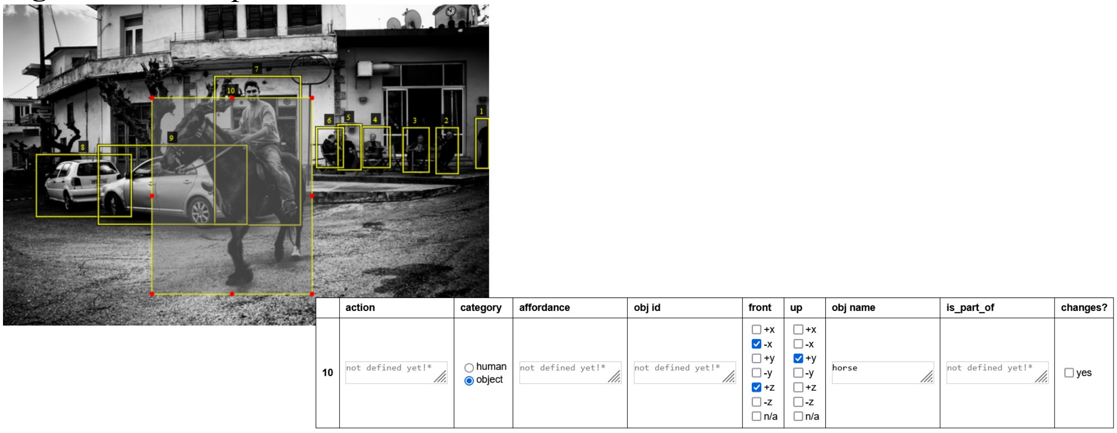

Figure 1b. Example of object annotation.

Figure 1. Example Annotations

Obj Category	Cohen's Kappa
apple	0.6028
bicycle	0.6105
bottle	0.6411
car	0.4234
chair	0.0
cup	0.6321
dog	0.0451
horse	0.4226
knife	0.4785
umbrella	-0.0080

Table S1. Calculated IAA between the image and text annotations. The low kappa values for some classes can be explained by the fact that the language in the caption may not capture the unique telic affordance that is present in the image (e.g., "standing under an umbrella"), so the caption annotation alone would mark this as G, which still allows T.

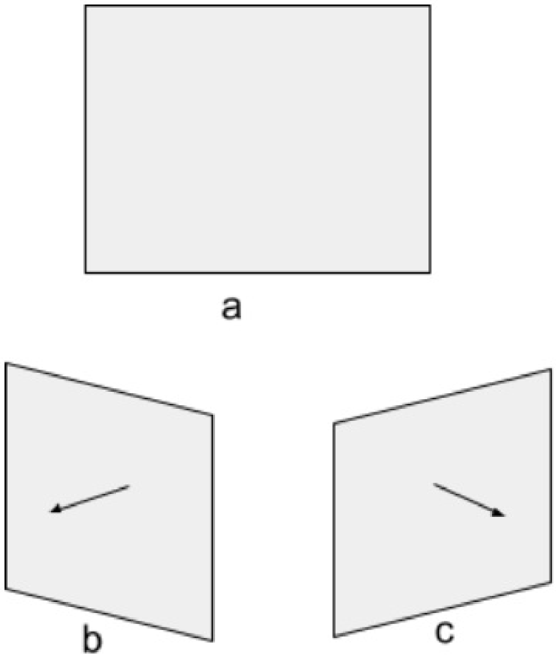

Figure S2. Example Object Orientation

1 ANNOTATION

Figure S2 shows few sample object orientations. The *front* orientations are (a) +z (b) -x+z (turned halfway towards the left) (c) +x+z (turned halfway towards the right) . Since the object has two pairs of identical edges (parallel edges), we can ignore the *up* orientations or mark it *n/a* in this case.

In Figure 1a, the human (inside the red dots - with id 7) exhibits actions no_interaction, no_interaction, ride with respect to objects with ids 8, 9 and 10 (Figure 1 in main paper). The affordances for the actions are None, None and telic respectively. The front side of the human is pointed towards the -x+z direction and the top in the +y direction. In Figure 1b, the object (the horse - object id 10) is oriented in the -x+z direction (front) and in the +y direction (top).

The calculated Inter-Annotator Agreement (IAA) values via Cohen's Kappa (Cohen, 1960) are listed in Table S1.

2 POSECONTRAST

Since PoseContrast uses ResNet for feature generation, we visualized a random subset of each 2 000 random images from HICO-DET and ObjectNet3D using t-SNE (Van der Maaten and Hinton, 2008; Van Der Maaten, 2014) (see Figure S3). We additionally analyzed the correct prediction as a function of object size (in pixels) and blur factor, but could not find any particular correlation (see Figure 4). Newer posture prediction models seem to handle unknown objects even better, but are currently not open source (Goodwin et al., 2022; Liu et al., 2022). Based on the underlying training data (e.g. CO3D (Reizenstein et al., 2021)), where the objects are again mainly in rest positions, it is unlikely to provide sufficient improvement for our application. Table S2 contains a small selection of pose datasets to illustrate the diversity of the data sets.

**Dataset**	**Domain**	**Obj Classes**	**Images / Videos**
300W<br>Sagonas et al. (2013)	Faces	1	600<br>Images
Animal-Pose<br>Cao et al. (2019)	Animals	5	4 000<br>Images
BEHAVE<br>Bhatnagar et al. (2022)	HOI	20	321<br>Videos
CO3D<br>Reizenstein et al. (2021)	Objects	50	1.5M<br>Images
COCO<br>Lin et al. (2014)	Human<br>Pose	1*	66 808*<br>Images
D3D-HOI<br>Xu et al. (2021)	HOI	8	256<br>Videos
IKEA<br>Lim et al. (2013)	Furniture	8	800<br>Images
KITTI-360<br>Liao et al. (2021)	Traffic	37	320 000<br>Images
Linemod<br>Hinterstoisser et al. (2012)	Houshold<br>Objects	15	1 100<br>Images
MPII<br>Andriluka et al. (2014)	Human<br>Pose	1	25 000<br>Images
NOCS<br>Wang et al. (2019)	Tabletop<br>Scenes	6	14 Real<br>300K AR
ObjectNet3D<br>Xiang et al. (2016)	Objects	100	90 000<br>Images
Objectron<br>Ahmadyan et al. (2021)	Objects	9	15K Videos<br>4M Images
Pascal3D+<br>Xiang et al. (2014)	Objects	12	36 000<br>Images
Pix3D<br>Sun et al. (2018)	Indoor	9	10 000<br>Images

Table S2. Selection of object orientation datasets with information about their size and domain coverage. COCO is a subset with pose information for the persons.

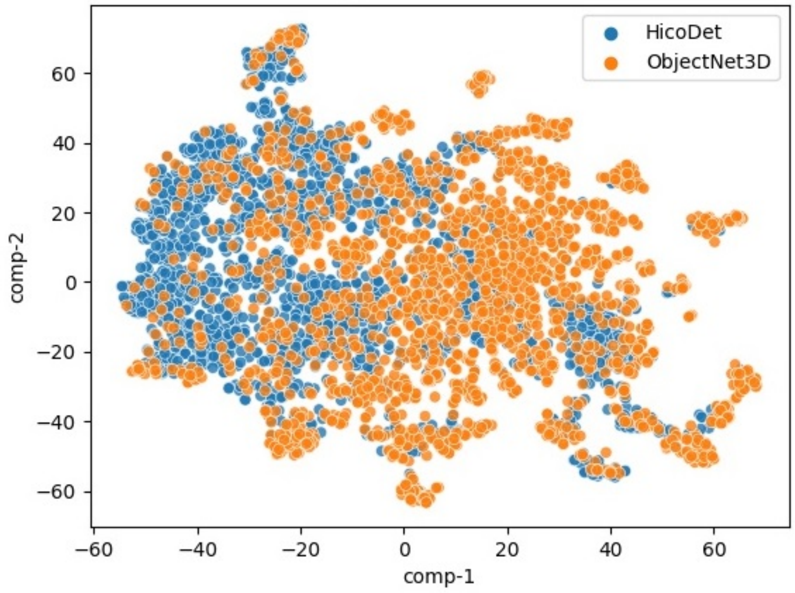

Figure S3. t-SNE visualization of ResNet features for 2 000 images each from ObjectNet3D and HICO-DET. While the densities of the two datasets differ significantly, ObjectNet3D finds complete overlap with HICO-DET (in two dimensions) as the number of samples increases.

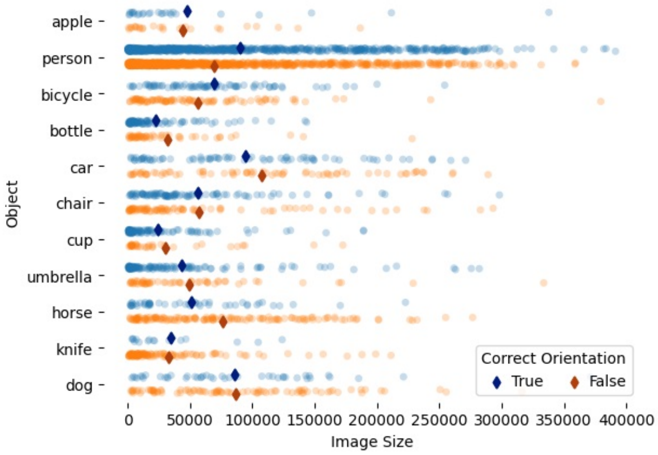

Figure 4a. object size (in pixel)

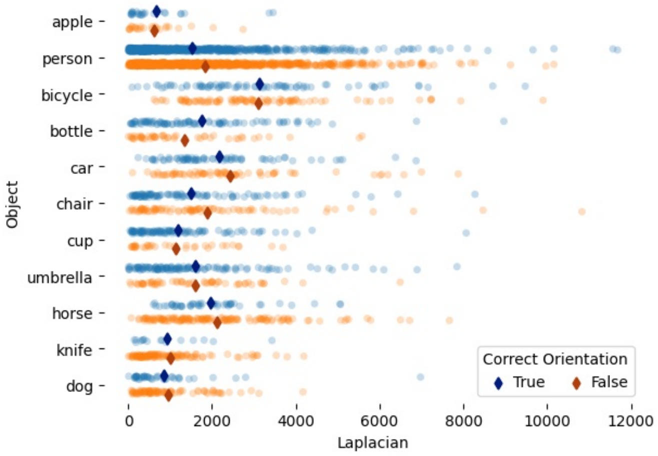

Figure 4b. blur

Figure 4. PoseContrast Object orientation determination considering image size **(A)** and blur **(B)**. Blur was calculated with the variance of Laplacian (Bansal et al., 2016). A higher value means a sharper image. Diamonds represent the mean.

3 AFFORDANCE UPT

The optimized hyperparameter are listed in Table S3. We used HDBSCAN (McInnes et al., 2017) to determine the main clusters for the t-SNE variant from Figure 5 from the main paper with HDBSCAN. The result is shown in Figure S7.

Figure 5 and 6 show the visualizations of the unary tokens. It can be seen that these probably represent mostly class information. And in Figure 6 it can be seen that only from the representation of the person no affordance can be derived either.

Figure 8 shows some more examples of boundary box distinguished between AffordanceUPT and HICO-DET.

Hyperparameter	Value
learning rate	1.3e-4
weight decay	4.7e-4
learning rate drop	82
gradient clip	0.18
loss alpha	0.25
loss gamma	0.85

Table S3. Hyperparameter for AffordanceUPT.

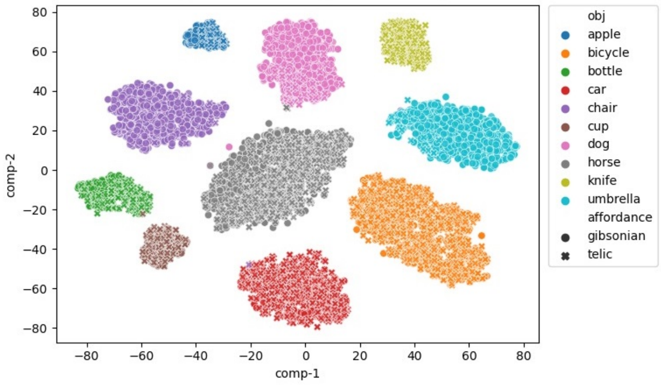

Figure 5a. t-SNE

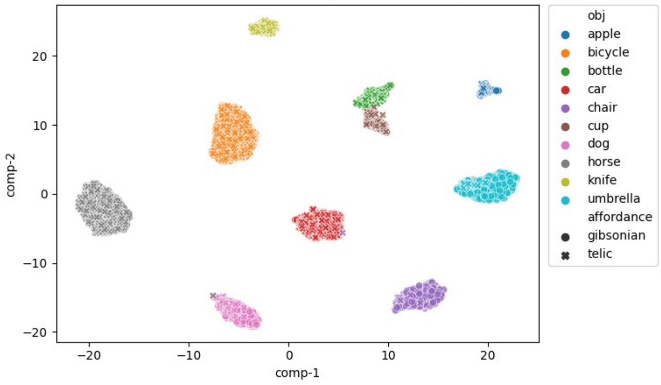

Figure 5b. PaCMAP

Figure 5. UPT Object Unary Token Visualization. Labeled according to their classes and in which affordance was determined for this object.

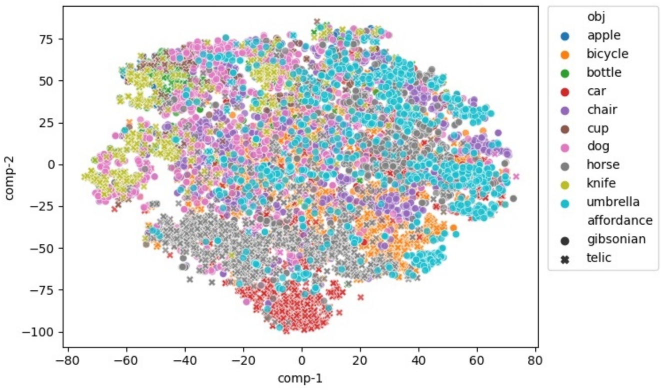

Figure 6a. t-SNE

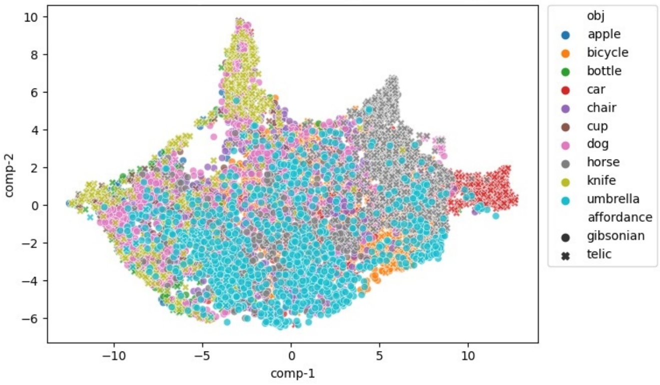

Figure 6b. PaCMAP

Figure 6. UPT Person Unary Token Visualization. Labeled according to their affordance and interacted object.

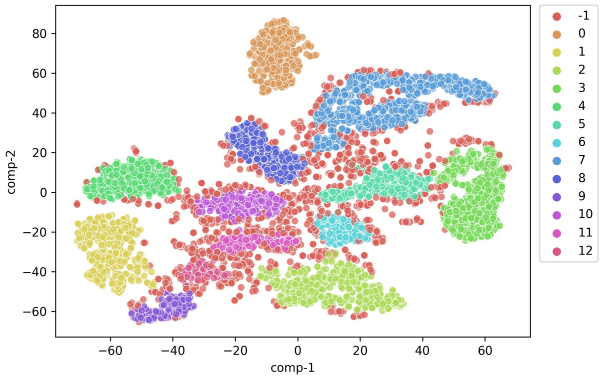

Figure S7. HDBSCAN results with *minimal cluster size*: 50 and *maximal cluster size*: 1000 on data shown in Figure 5 of the main paper. Homogeneity: 0.603; Completeness: 0.675; V-measure: 0.637; Adjusted Rand Index: 0.436; Adjusted Mutual Information: 0.634. Points with label -1 were classified as noise.

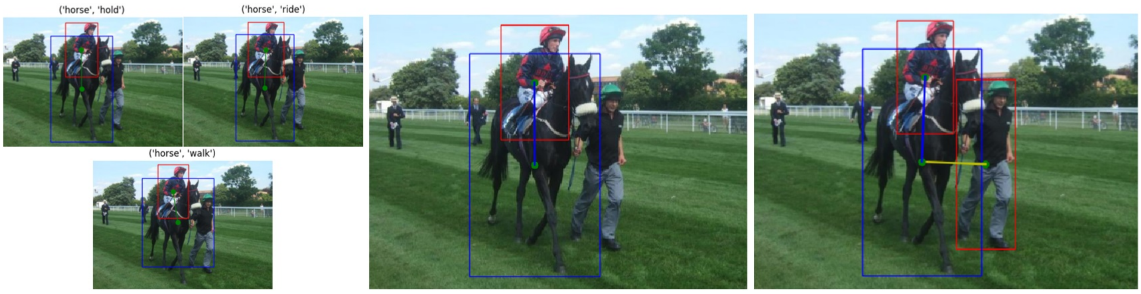

Figure 8a. The right person is not part of the HICO-DET annotation.

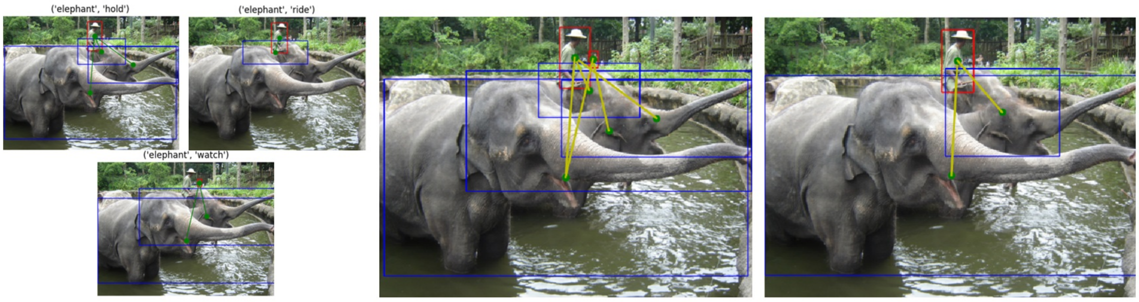

Figure 8b. Different bounding boxes for the same objects, which are unmergable.

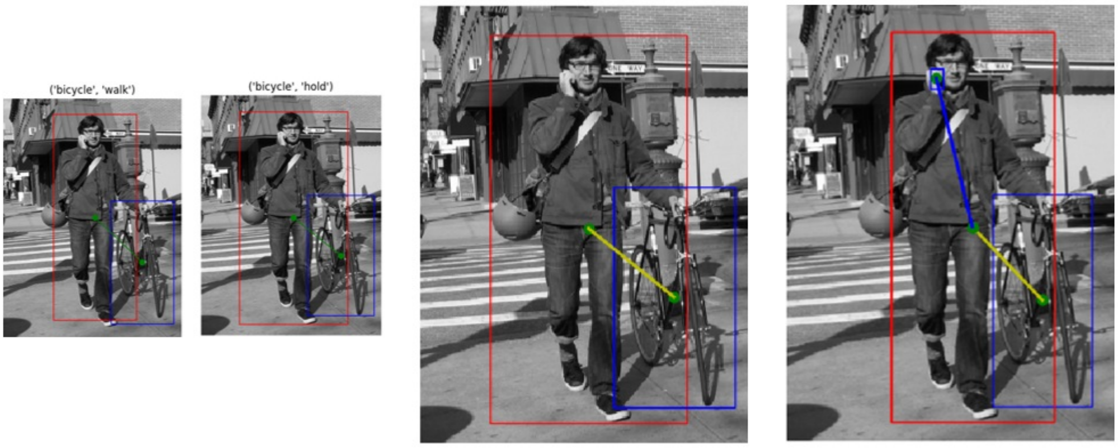

Figure 8c. Cell phone is not part of the HICO-DET annotation.

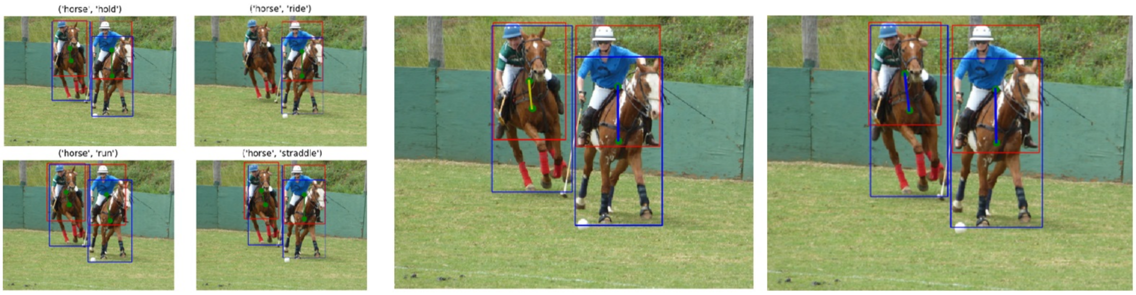

Figure 8d. The rear left horse was not annotated as a “ride”, which is why it shows up in the dataset as Gibsonian.

Figure 8. Various error cases of AffordanceUPT based on HICO-DET annotations. The left images are the original HICO-DET annotations, the middle images are the merged variants with Gibsonian (yellow) and telic (blue) connections and the right images are the predictions by AffordanceUPT.

REFERENCES

Ahmadyan, A., Zhang, L., Ablavatski, A., Wei, J., and Grundmann, M. (2021). Objectron: A large scale dataset of object-centric videos in the wild with pose annotations. *Proceedings of the IEEE Conference on Computer Vision and Pattern Recognition*

Andriluka, M., Pishchulin, L., Gehler, P., and Schiele, B. (2014). 2d human pose estimation: New benchmark and state of the art analysis. In *IEEE Conference on Computer Vision and Pattern Recognition (CVPR)*

Bansal, R., Raj, G., and Choudhury, T. (2016). Blur image detection using laplacian operator and open-cv. In *2016 International Conference System Modeling & Advancement in Research Trends (SMART)* (IEEE), 63–67

Bhatnagar, B. L., Xie, X., Petrov, I., Sminchisescu, C., Theobalt, C., and Pons-Moll, G. (2022). Behave: Dataset and method for tracking human object interactions. In *IEEE Conference on Computer Vision and Pattern Recognition (CVPR)* (IEEE)

Cao, J., Tang, H., Fang, H.-S., Shen, X., Lu, C., and Tai, Y.-W. (2019). Cross-domain adaptation for animal pose estimation. In *The IEEE International Conference on Computer Vision (ICCV)*

Cohen, J. (1960). A coefficient of agreement for nominal scales. *Educational and psychological measurement* 20, 37–46

Goodwin, W., Vaze, S., Havoutis, I., and Posner, I. (2022). Zero-shot category-level object pose estimation. *arXiv preprint arXiv:2204.03635*

Hinterstoisser, S., Lepetit, V., Ilic, S., Holzer, S., Bradski, G., Konolige, K., et al. (2012). Model based training, detection and pose estimation of texture-less 3d objects in heavily cluttered scenes. In *Asian conference on computer vision* (Springer), 548–562

Liao, Y., Xie, J., and Geiger, A. (2021). KITTI-360: A novel dataset and benchmarks for urban scene understanding in 2d and 3d. *arXiv preprint arXiv:2109.13410*

Lim, J. J., Pirsiavash, H., and Torralba, A. (2013). Parsing IKEA Objects: Fine Pose Estimation. In *ICCV*

Lin, T.-Y., Maire, M., Belongie, S., Hays, J., Perona, P., Ramanan, D., et al. (2014). Microsoft coco: Common objects in context. In *European conference on computer vision* (Springer), 740–755

Liu, Y., Wen, Y., Peng, S., Lin, C., Long, X., Komura, T., et al. (2022). Gen6d: Generalizable model-free 6-dof object pose estimation from rgb images. *arXiv preprint arXiv:2204.10776*

McInnes, L., Healy, J., and Astels, S. (2017). hdbscan: Hierarchical density based clustering. *The Journal of Open Source Software* 2. doi:10.21105/joss.00205

Reizenstein, J., Shapovalov, R., Henzler, P., Sbordone, L., Labatut, P., and Novotny, D. (2021). Common objects in 3d: Large-scale learning and evaluation of real-life 3d category reconstruction. In *Proceedings of the IEEE/CVF International Conference on Computer Vision*. 10901–10911

Sagonas, C., Tzimiropoulos, G., Zafeiriou, S., and Pantic, M. (2013). A semi-automatic methodology for facial landmark annotation. In *Proceedings of the IEEE conference on computer vision and pattern recognition workshops*. 896–903

Sun, X., Wu, J., Zhang, X., Zhang, Z., Zhang, C., Xue, T., et al. (2018). Pix3d: Dataset and methods for single-image 3d shape modeling. In *IEEE Conference on Computer Vision and Pattern Recognition (CVPR)*

Van Der Maaten, L. (2014). Accelerating t-sne using tree-based algorithms. *The journal of machine learning research* 15, 3221–3245

Van der Maaten, L. and Hinton, G. (2008). Visualizing data using t-sne. *Journal of machine learning research* 9

Wang, H., Sridhar, S., Huang, J., Valentin, J., Song, S., and Guibas, L. J. (2019). Normalized object coordinate space for category-level 6d object pose and size estimation. In *The IEEE Conference on Computer Vision and Pattern Recognition (CVPR)*

Xiang, Y., Kim, W., Chen, W., Ji, J., Choy, C., Su, H., et al. (2016). Objectnet3d: A large scale database for 3d object recognition. In *European Conference Computer Vision (ECCV)*

Xiang, Y., Mottaghi, R., and Savarese, S. (2014). Beyond pascal: A benchmark for 3d object detection in the wild. In *IEEE Winter Conference on Applications of Computer Vision (WACV)*

Xu, X., Joo, H., Mori, G., and Savva, M. (2021). D3d-hoi: Dynamic 3d human-object interactions from videos. *arXiv preprint arXiv:2108.08420*
